# The power of primary health care

**DOI:** 10.1080/02813432.2020.1841507

**Published:** 2020-12-30

**Authors:** Jon Steinar Jonsson, Emil Larus Sigurdsson

**Affiliations:** Department of Family Medicine, University of Iceland, Reykjavík, Iceland; Development Centre for Primary Health Care in Iceland

When the Covid-19 pandemic began last winter, primary health care (PHC) in many countries faced entirely new challenges. The role of the PHC in this huge project was large but in fact it differed somewhat between countries. The gatekeeping role was of enormous importance, making early diagnoses and assisting people who were worried about this pandemic. Also, among the most crucial roles of PHC was to protect the hospitals from being overloaded [1].

In Iceland, the activities of the PHC were completely transformed. Considerable changes were made in the form of communication with patients during the pandemic. More indirect communication took place *via* telephone, web consultation and telemedicine. The experience gained from these means of communication is generally positive and will undoubtedly have an impact on how the PHC will conduct its future relations with its clients. This is in line with the tendency seen in many other countries [2].

Another thing that is interesting to observe is what effect enforced isolation of those infected and a quarantine of those exposed will have on the frequency of other infections and in the use of antibiotics, especially among children. Antibiotic overuse and particularly the over prescribing of antibiotics for children being of extreme importance in PHC [3,4]. It seems that in Iceland, the use of antibiotics among children decreased during the first wave of covid-19, from March to May 2020 [2]. There might be two reasons for this. Firstly, simply it could have been due to fewer infections, and secondly, this could be the effect of less access to healthcare that was spending most of its time dealing with Covid-19. The 2-m social distancing rule was mandatory and hand washing and limiting public and private gatherings no doubt had an effect as well.

Therefore, the question is whether we should spend more effort to prevent infection from spreading in children by in order to reduce the use of antibiotics. Is more hand washing, spraying and similar measures something that has come to be?

There are huge variations in the effects of Covid-19 on individuals and families. In some cases, this has caused considerable problems and even increased isolation. The ban on visits to nursing homes has in many cases caused great discomfort to the residents' relatives. On the other hand, restrictions on travel between countries have had a positive effect on domestic travel. Thus, Icelanders have significantly reduced travel abroad and instead supported the domestic tourism industry. This means that people get better acquainted with their own country and at the same time spend far less foreign currency abroad.

At the end of July, another wave of epidemic started with an increased number of cases being detected in many countries. How and when the Covid-19 pandemic will come to an end is still not known but persistent symptoms are already well-known among patients who have been infected with Covid-19, even among patients with mild symptoms of the infection. Post-acute covid-19 is an emerging task for PHC [5]. Approximately 10% of Covid-19 patients can be expected to persist with symptoms three weeks after being diagnosed with Covid-19. These symptoms vary widely and among the most common are cough, fatigue, low grade fever, shortness of breath and depression. As the Covid-19 is a multisystem disease, with thromboembolic being one, some of these patients might have develop a life-threatening disease such as pulmonary embolism. Thus, this task is very challenging. These patients need comprehensive whole-patient assessment, involving a team of different professionals including general practitioners, nurses, psychologists, social workers, physiotherapists and others.

With PHC being occupied with the Covid-19, the danger is that some urgent tasks could remain unfinished. An increased unemployment rate and more severe financial problems, together with mental illnesses in the next year will increase the workload on the PHC even more. So far, the PHC has once more shown its strength and resilience in meeting this new challenge. Hopefully, it will be able to provide its patients with an effective and meaningful health-care service when it comes to post-acute Covid symptoms.

## References

[1] https://apps.who.int/iris/handle/10665/331921. 2020.

[2] Thulesius H. Increased importance of digital medicine and eHealth during the Covid-19 pandemic. Scand J Prim Health Care. 2020;38(2):105–106. 32484725 10.1080/02813432.2020.1770466 PMC8570755

[3] Laura Trolle S, Lars B, Volkert S, et al. Quality assessment in general practice: diagnosis and antibiotic treatment of acute respiratory tract infections. Scand J Prim Health Care. 2018;36(4):372–379. 30296885 10.1080/02813432.2018.1523996 PMC6381521

[4] Marieke B L, Jan Y V, Roos C, et al. Point-of-care CRP matters: normal CRP levels reduce immediate antibiotic prescribing for acutely ill children in primary care: a cluster randomized controlled trial. Scand J Prim Health Care. 2018;36(4):423–436. 30354904 10.1080/02813432.2018.1529900 PMC6381547

[5] Greenhalgh T, Knight M, A'Court C, et al. Management of post-acute covid-19 in primary care. BMJ. 2020;370:m3026. 32784198 10.1136/bmj.m3026

